# Antimicrobial resistance of *Vibrio* spp. from the coastal California system: discordance between genotypic and phenotypic patterns

**DOI:** 10.1128/aem.01808-24

**Published:** 2025-02-03

**Authors:** Peter J. Sebastian, Cory Schlesener, Barbara A. Byrne, Melissa Miller, Woutrina Smith, Francesca Batac, Caroline E. C. Goertz, Bart C. Weimer, Christine K. Johnson

**Affiliations:** 1 EpiCenter for Disease Dynamics, One Health Institute, School of Veterinary Medicine, University of California, Davis 660406 , Davis, California, USA; 2 Karen C. Drayer Wildlife Health Center, One Health Institute, School of Veterinary Medicine, University of California, Davis 502227 , Davis, California, USA; 3 Department of Population Health and Reproduction, 100K Pathogen Genome Project, School of Veterinary Medicine, University of California, Davis 240471 , Davis, California, USA; 4 Department of Pathology, Microbiology and Immunology, School of Veterinary Medicine, University of California, Davis 240470 , Davis, California, USA; 5 Marine Wildlife Veterinary Care and Research Center, California Department of Fish and Wildlife, Santa Cruz, California, USA; 6 Alaska SeaLife Center, Seward, Alaska, USA; INRS Armand-Frappier Sante Biotechnologie Research Centre, Laval, Canada

**Keywords:** antimicrobial resistance, antibiotic resistance, genomic epidemiology, environmental microbiology, vibriosis, sea otter, *Enhydra lutris*

## Abstract

Antimicrobial resistance in *Vibrio* species poses risks to both human and marine mammal health. Whole genome sequencing of *Vibrio* spp. can be utilized to screen for antimicrobial resistance genes and allelic variants to provide mechanistic insights in ways that PCR screening and phenotypic interpretation cannot. Our goals were to (i) characterize antimicrobial resistance patterns of *Vibrio* spp. pathogens isolated from southern sea otters (*Enhydra lutris nereis*), northern sea otters (*Enhydra lutris kenyoni*), and environmental samples from the central California coast using whole genome sequencing, and (ii) compare the presence of antimicrobial resistance genes with phenotypic interpretation from antibiotic susceptibility testing. Unexpectedly, genomic classification identified an understudied species, *Vibrio diabolicus*, in sea otter and environmental isolates that were previously identified as *Vibrio alginolyticus*. A total of 489 *Vibrio* spp. isolates were sequenced, and frequently detected antimicrobial resistance genes included multidrug efflux pumps and genes associated with resistance to ß-lactams and tetracyclines. Genes associated with resistance to fluoroquinolones, aminoglycosides, chloramphenicol, and sulfonamides were uncommon. Sea otter isolates were phenotypically susceptible to tetracycline despite carrying genes *tet34* and *tet35*. Both between- and within-species variations in ampicillin resistance were observed despite the ubiquitous presence of *bla*_CARB_ genes in *V. alginolyticus*, *V. diabolicus*, and *Vibrio parahaemolyticus*. Discordance between phenotypic and genotypic ampicillin resistance was especially noted for *V. parahaemolyticus* and was partially attributed to the allelic variation of the *bla_CARB_* genes. Tetracyclines and fluoroquinolones, but not ß-lactams, are likely to be effective treatments for vibriosis in sea otters.

**IMPORTANCE:**

Vibriosis (infection with non-cholera *Vibrio* spp.) is the most common seafood-borne illness globally, with major impacts on public health, food security, and wildlife health. Potential treatments of antimicrobial-resistant *Vibrio* spp. in humans, aquaculture, and marine wildlife rehabilitation are complicated by current diagnostic challenges regarding bacterial species identification and interpretation of antimicrobial resistance patterns. Unexpected detection of previously misidentified *Vibrio diabolicus* in sea otters suggests that a broader taxonomic group of *Vibrio* infect sea otters than previously described. We also determined that the presence of ß-lactamase genes alone in sea otter isolates does not necessarily correlate with an ampicillin-resistant phenotype, likely due to deleterious amino acid substitutions in certain *bla*_CARB_ alleles. Continued monitoring of *Vibrio* spp. phenotypes and genotypes in sea otters is warranted to observe biologically relevant changes in antimicrobial resistance.

## INTRODUCTION

The *Vibrio* genus is a diverse group of gram-negative, rod-shaped bacteria with two circular chromosomes that inhabit primarily warm, brackish marine, and estuarine environments. There are over 100 *Vibrio* species, including many that impact marine wildlife, and several that are implicated in human illness. Vibriosis (infection with non-*cholerae Vibrio* spp. and marine-inhabiting non-O1/O139 *Vibrio cholerae*) accounts for approximately 80,000 human illnesses in the USA each year (1). *Vibrio parahaemolyticus* is the most common bacterial cause of shellfish-related illness, resulting in gastroenteritis and sepsis in severe cases. *Vibrio alginolyticus*, one of the top four most reported *Vibrio* pathogens, is primarily associated with wound and ear infections following seawater exposure (1). *Vibrio diabolicus*, first isolated near a deep-sea vent, has since been identified in coastal habitats, is closely related to *V. alginolyticus*, and in some cases has been misclassified as *V. alginolyticus* (2, 3).

*Vibrio* spp. are frequently detected in benthic invertebrates consumed by marine mammals and humans (4–7). Potentially pathogenic *Vibrio* spp., primarily non-O1/O139 *V. cholerae*, *V. parahaemolyticus*, and *V. alginolyticus*, have been found in healthy (8–12), clinically ill, and dead marine mammals (13–16) along the North American Pacific coast. Sea otters have been proposed as highly sensitive ecological bioindicators; study of disease in these animals can highlight the presence of multiple pathogens and contaminants in the near-shore environment (17, 18). Sea otters are likely to be an effective host to model antimicrobial resistance (AMR) in *Vibrio* spp. infections.

Unique biological attributes that make sea otters outstanding as putative environmental sentinels of AMR in *Vibrio* spp. include their relatively small near-shore home range and their consumption of high volumes of shellfish and benthic invertebrates, including clams, mussels, and other species that are also consumed by humans and are important sources of human vibriosis (17, 18). Multiple other disease processes with public health importance have been successfully modeled in southern sea otters (*Enhydra lutris nereis*) including *Microcystis aeruginosa*, *Toxoplasma gondii*, and domoic acid toxicosis (19–21). *Vibrio* infections in northern sea otters (*Enhydra lutris kenyoni*) from Alaska have been proposed as indicators of climate change, especially *V. parahaemolyticus,* which has begun to be detected with Alaskan coastal water temperatures rising past 15°C (10). *Vibrio* infections in sea otters are thought to occur as secondary infections or sequelae to traumatic injury, which may require effective antimicrobial therapy during rehabilitation (8, 16).

While *Vibrio* spp. are traditionally susceptible to most clinically relevant antimicrobials including tetracyclines, fluoroquinolones, and third-generation cephalosporins, there has been an increase in the awareness and incidence of antimicrobial-resistant *Vibrio* spp. isolates (22, 23). Characterization of AMR in *Vibrio* spp. from southern sea otters is limited with *V. cholerae* (*n* = 3) isolates interpreted as having clindamycin-resistant, tylosin-resistant, and erythromycin intermediate phenotypes, and *V. parahaemolyticus* (*n* = 15) isolates interpreted as exhibiting variable phenotypes including either ampicillin intermediate or erythromycin-, cefazolin-, clindamycin-, and tylosin-resistant phenotypes (8). Characterization of AMR phenotypes of *Vibrio* spp. across diverse sources within coastal communities such as marine wildlife, marine invertebrates, and environmental samples could help protect human health, facilitate assessment of epidemiological patterns and trends, and optimize care for stranded marine mammals.

Studies of AMR in *Vibrio* spp. typically involve either antimicrobial susceptibility testing to determine phenotype, or amplification and genotypic assessment of target AMR genes (5, 24–26). Study designs are often limited by the limited number of antibiotics tested, or the number and specificity of the AMR gene primers used. As whole genome sequencing becomes more cost-effective, it is increasingly used to detect both known and unknown AMR genes and determine allelic variants associated with phenotypic resistance (27, 28). However, discordance between genotypic and phenotypic AMR predictions is common for *Vibrio* species. For example, *Vibrio* spp. from Norway carrying ß-lactamase genes were still susceptible to cephalosporins (29).

Given the multiple gaps in knowledge around AMR patterns of *Vibrio* spp., our objectives were to (i) improve the phenotypic and genotypic characterization of AMR in *Vibrio* spp. isolated between 2000 and 2019 in an understudied putative bioindicator species in the near-shore environment shared with humans and (ii) examine the associations and discordance between genome-based detection of AMR genes with the phenotypic patterns observed from antibiotic susceptibility testing. We hypothesized that *Vibrio* spp. isolates from sea otters and their environment will carry AMR genes with treatment implications for vibriosis and that there will be discordance between phenotypic AMR patterns and their putative genetic determinants.

## MATERIALS AND METHODS

### Sample collection

*Vibrio* spp. isolates stored at −80°C at the University of California, Davis – Veterinary Medical Teaching Hospital from 2000 to 2019 were used for whole genome sequencing. Isolates originated from previous California-based projects including avian feces from wildlife hospital admissions and wild *Larus* spp. gulls (9, 30); live-sampled and necropsied southern sea otters (8, 9, 14, 31); shellfish samples from *Mytilus* spp. mussels and pismo clams (*Tivela stultorum*) (32); water samples collected near Big Sur, California; environmental samples (water, sediment, algae, seagrass, and kelp swabs) from Elkhorn Slough, California; and three water samples from the Washington State coastline. Additional *Vibrio* spp. isolates were collected from live and dead northern sea otters in Alaska from 2004 to 2015 (10, 16). Cary-Blair transport media without added antibiotics was used (Hardy Diagnostics, Santa Maria, CA, USA), and water samples were filtered and then placed into alkaline peptone water for an additional incubation step 18–24 hours before subculture on thiosulfate-citrate-bile salts-sucrose (TCBS) agar at 35°C without CO_2_ (14).

### Whole genome sequencing and genome assembly

Frozen *Vibrio* spp. isolates banked on Microbank beads (Pro-Lab Diagnostics, Fisher Scientific, Round Rock, TX, USA) were plated on 5% sheep blood agar (Biological Media Services, University of California, Davis; Hardy Diagnostics, Santa Maria, CA, USA) at 37°C with 5% CO_2_ for 24 hours. Isolates were clinically speciated using biochemical testing (API20E, BioMerieux, Durham, NC, USA), and a subset of isolates were confirmed by matrix-assisted laser desorption-ionization mass spectrometry (MALDI-ToF; Bruker Daltonics, Fremont, CA, USA) using the Bdal library in MBT Compass 4.1 (33). DNA was extracted using the spin column protocol from the QiaAMP UCP Pathogen extraction kit with the additional mechanical pre-lysis protocol using small pathogen lysis tubes (Qiagen, Hilden, Germany). Due to shipping/manufacturing delays, some extractions were performed using the Wizard genomic DNA purification kit (Promega Corporation, Madison, WI, USA). The quality and yield of genomic DNA were verified using a 2200 TapeStation with genomic DNA ScreenTape (Agilent Technologies, Santa Clara, CA, USA) (34).

Whole genome sequencing was performed by the Weimer laboratory at the University of California, Davis, as part of the 100K Pathogen Genome Project (http://www.genomes4health.org/) (7, 34–38). Briefly, whole genome sequencing was performed using Illumina HiSeq XTEN with PE 150 plus index read (Illumina, San Diego, CA, USA). Libraries were constructed from fragmented DNA (1 µg) using the HTP library preparation kit (Kapa Biosystems, Wilmington, MA, USA) (33, 39). Adapters and Illumina standards were trimmed from paired-end FASTQ files using Trimmomatic (v0.39) (40) to remove TruSeq universal adapters following previously described parameters (41) before quality control of reads using FastQC (v0.11.9) (42). Genomes were assembled from trimmed reads using Shovill (v1.0.4) (43), and genes were annotated with Prokka (v1.14.6) (44). Shovill, which improves the speed of SPAdes assembly, and Prokka were chosen for their widespread utility to be comparable with other microbial genomic studies.

Genome quality was assessed using FastQC and CheckM (v1.1.2) (45); genomes with <90% genome completeness, >5% contamination, >300 contigs, or <20× estimated genome coverage were removed from the analysis. Taxonomic classification to the species level was confirmed with Kraken2 (v2.0.8) used with a RefSeq microbial genomes database (downloaded 5 May 2021) constructed with standard tools and microbe categories followed by Bayesian re-estimation of Kraken2 hits with Bracken (v2.6.1) (46, 47). An all-against-all genomic similarity comparison between pairwise genomes was visualized by MinHash sketches using Sourmash (v3.2.3) with a k-mer size of 31 and a genome sketch size of 100,000 k-mers per megabase (48, 49). Genomes with Jaccard Similarity Index scores equal to 1 were considered genetically near-identical or identical and were condensed when these isolates arose from the same sample or individual host. Multilocus sequence typing (MLST) was performed using *Vibrio* species schemes from PubMLST (50, 51) run through mlst software (52). The software was run with a four loci *Vibrio* spp. multilocus sequence analysis (MLSA) scheme (53), and run a second time using autodetection to determine the most appropriate scheme for each genome including schemes for non-O1/O139 *V. cholerae* and *V. parahaemolyticus* (54, 55).

### Antimicrobial resistance gene detection

Contigs from assembled genomes were screened for AMR genes using ABRicate (v1.0.1) (T. Seemann, https://github.com/tseemann/abricate) to search the following databases downloaded on 17 March 2021: Comprehensive Antibiotic Resistance Database (CARD, https://card.mcmaster/ca) (56), ResFinder (57–60), MegaRes (61), NCBI AMRFinder (62), and ARG-ANNOT (63). Gene hits were compiled from all databases, and minimum cutoffs of 70% identity and 70% coverage were used to remove spurious gene hits. Allelic variants of ß-lactamase genes were called using snippy (v4.6.0, https://github.com/tseemann/snippy) (64) with the following reference isolates: BCW_12327 (*V. parahaemolyticus*, *bla*_CARB-21_, locus tag GGOADLPD_00464), BCW_11123 (*V. cholerae* non-O1/O139, *bla*_CARB-7_, locus tag EHPCMFLH_03524), and BCW_12271 (*V. alginolyticus*, *bla*_CARB-42_, locus tag KHCBNDJJ_00127, *ampC*, locus tag KHCBNDJJ_00781). InterproScan was used to predict the active site and protein domain for *bla*_CARB_ genes in *V. parahaemolyticus* (65, 66).

### Antimicrobial susceptibility testing

Prior to whole genome sequencing, a subset of 165 southern sea otter isolates (*V. alginolyticus* = 37, *V. diabolicus* = 20, *V. cholerae* = 46, *V. parahaemolyticus* = 62) were opportunistically selected for antimicrobial susceptibility using a microbroth dilution method based on Clinical Laboratory Standards Institute guidelines (67) using a standardized National Antimicrobial Resistance Monitoring System (NARMS Gram negative) panel (Sensititre, Thermo Fisher Scientific, Waltham, MA, USA). Only a subset of samples could be tested, thus we focused on southern sea otter isolates because of conservation interest as a threatened species. Briefly, *Vibrio* spp. isolates were inoculated in brain heart infusion broth (Biological Media Services, University of California, Davis, CA, USA) and incubated at 35°C under room atmosphere for 2–4 hours. Growth was adjusted using a nephelometer (Sensititre, Thermo Fisher Scientific) to the equivalent a 0.5 McFarland standard in 0.85% NaCl, and 10 mL of saline solution was inoculated into cation-adjusted Mueller-Hinton broth (Sensititre, Thermo Fisher Scientific). Sensititre trays (Sensititre, NARMS Gram negative plate CMV3AGNF, Thermo Fisher Scientific) were inoculated with 50 mL of the Mueller-Hinton suspension before being sealed and incubated for 18–24 hours at 35°C under room atmosphere before reading. Each panel tested susceptibility to the following 13 antibiotics: ampicillin, amoxicillin/clavulanic acid, ceftriaxone, ceftiofur, cefoxitin, ciprofloxacin, nalidixic acid, chloramphenicol, sulfisoxazole, sulfamethoxazole/trimethoprim, tetracycline, azithromycin, and gentamicin. Each panel included positive and negative controls, and weekly quality controls were conducted with ATCC strains of *Escherichia coli, Staphylococcus aureus, Pseudomonas aeruginosa,* and *Enterococcus faecalis*.

Interpretation of the minimum inhibitory concentrations (MICs) was based on Clinical Laboratory Standards Institute guidelines for *Vibrio* spp. using the intermediate and resistant breakpoints validated from human sources (67). While no interpretations currently exist for isolates from sea otters, a designation of intermediate was used to indicate the observed MIC (microgram per milliliter) is associated with an uncertain therapeutic effect, whereas the resistant designation was associated with a likelihood of therapeutic failure in human infections. For sulfisoxazole, chloramphenicol, and nalidixic acid, the breakpoints have only been validated for *V. cholerae* but were used here as a best estimate for non-*cholerae Vibrio* spp. (67). No clinical breakpoints were available for ceftiofur and ceftriaxone.

### Statistical analysis

Minimum inhibitory concentrations from antibiotic susceptibility tests were log2 transformed to linearize the data, and the log2 of the MIC was compared between *Vibrio* species for select antibiotics using Kruskal-Wallis tests and pairwise Wilcoxon rank-sum tests with a Benjamini and Hochberg correction for significant results. The proportion of isolates categorized as intermediate or resistant for each antimicrobial was compared between species using univariate logistic regression. Univariate logistic regression was used to investigate the presence/absence of AMR genes with categorical variables including *Vibrio* species, and within-species comparisons of sex (male, female, unknown), age (adults/aged adults, juveniles/subadults, unknown), year, season, and sample types (environmental, southern sea otter, and northern sea otter).

The presence of AMR genes or specific *bla*_CARB_ allelic variants was compared to antimicrobial susceptibility test susceptible versus intermediate/resistant interpretations using Chi-squared tests for a subset of 158 isolates that also passed sequencing quality control metrics (35 *V*. *alginolyticus*, 44 *V*. *cholerae*, 18 *V*. *diabolicus*, and 61 *V*. *parahaemolyticus*). For antimicrobials with a MIC below resistant breakpoints, Wilcoxon rank-sum tests were used to compare the log2 of the MIC with the presence of AMR genes, or alternatively, a Chi-squared test was used for antimicrobials with only two MIC values. The above analyses were performed in R (v4.0.3). SatScan software (v10.0) was used to investigate spatial patterns of AMR gene detection and phenotypic patterns of AMR based on MIC data. Clustal Omega (https://www.ebi.ac.uk/Tools/msa/clustalo/) was used for multiple sequence alignment of *bla*_CARB_ genes across isolates (68). Outputs from Clustal Omega were imported to JalView (v.2.11.2.0) for data visualization (69).

## RESULTS

### Species identification and genome similarity

A total of 489 *Vibrio* spp. isolates passed quality control metrics, and a large majority (410; 84%) showed consensus between the genomic species identity and the identity assigned using phenotypic methods in the clinical laboratory (Fig. S1). The biggest discrepancy between clinical and genomic species identification was that the clinic databases did not include *V. diabolicus*, which was clinically identified as either *V. alginolyticus* (*n* = 54) or *V. parahaemolyticus* (*n* = 3). Additionally, 11 *V*. *parahaemolyticus* genomes were clinically identified as *V. alginolyticus*. Discordance also occurred with species considered nonpathogenic to humans including isolates identified as *Vibrio ziniensis* and *Vibrio anguillarum* by genomics but identified as *Vibrio diazotrophicus* (*n* = 4) or *Vibrio aestuarianus* (*n* = 2), respectively, in the clinic. The autodetection of the most appropriate PubMLST scheme resulted in the consistently correct identification of *V. cholerae* and *V. parahaemolyticus* genomes but not classification of sequence type. An appropriate MLST scheme was not available for nonpathogenic *Vibrio* spp., nor did this scheme differentiate *V. diabolicus* from *V. alginolyticus* (Data Set S1).

**Fig 1:**
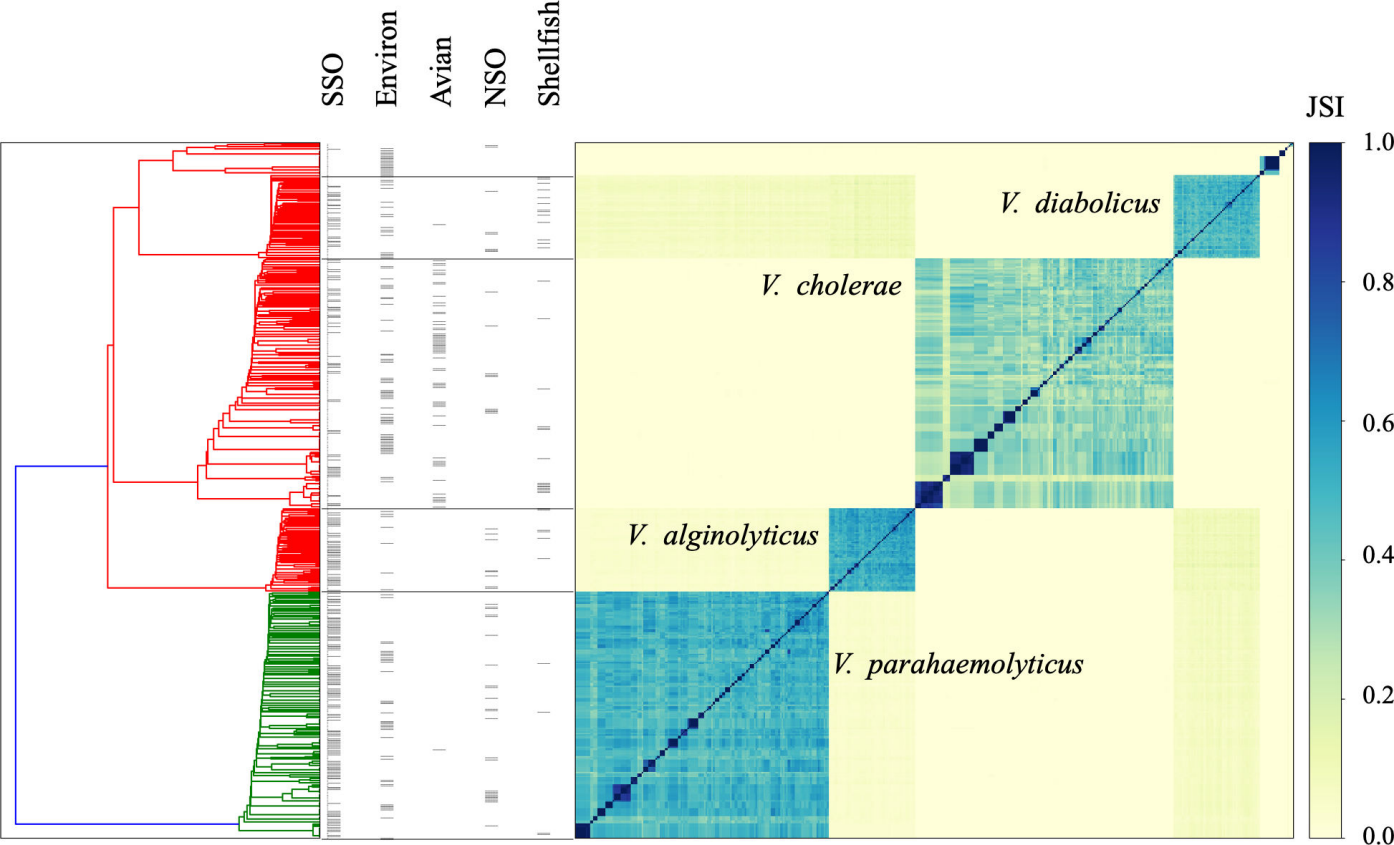
All-against-all comparative analysis of the similarity between 489 *Vibrio* species genomes collected between 2000 and 2019 from various sources in coastal Alaska, California, and Washington confirms genomes clustered by their genomic species identification. Within the four most frequently isolated species (labeled), there was both large genomic diversity and small clusters of near-identical or identical genomes. Darker blue colors on the heatmap indicate higher Jaccard Similarity Index (JSI) values and higher similarity between genomes while lighter yellow colors indicate lower similarity between genomes. Genomes are annotated by source from the most frequent to the least frequent: southern sea otters (SSO), environment, avian, northern sea otters (NSO), and shellfish.

The all-against-all genome comparative analysis indicated that all genomes were appropriately clustered by the whole genome species identification method (Fig. 1), further validating the genomic method as a higher-resolution method of species identification than clinical methods. Visualization of the pangenome rarefaction curves for *V. alginolyticus* (14,907 unique genes), *V. cholerae* (16,569 unique genes), *V. diabolicus* (15,152 unique genes), and *V. parahaemolyticus* (19,132 unique genes) indicated open pangenomes for all species (Fig. S2). Relatively low pairwise genomic similarity within each species indicated considerable within-species genomic diversity that was supported by the low contribution of the core genome to the total pangenome (e.g., 18.7% in *V. parahaemolyticus*).

Pairwise genome similarity comparisons also identified near-identical or identical genomes. Genomes were categorized as duplicates (*n* = 45) and excluded from further analysis if another near-identical or identical genome (i) originated from the same sample with multiple colonies sub-cultured, or (ii) was collected from a different tissue from the same host (Table 1). The remaining 444 genomes that were used in subsequent analyses consisted of 65 clusters of between 2 and 10 near-identical or identical genomes and 244 singleton genomes that were different from all other isolates, for a combined 309 unique genome types.

**TABLE 1:** Comparison of the number of genomes in each *Vibrio* species sampled from various sources in coastal Alaska, California, and Washington between 2000 and 2019 before and after removal of duplicates^*a*^

*Vibrio* species	Starting genomes	Duplicates removed	Remaining genomes	Clusters (size range)	Singletons	Unique genomes
*V. alginolyticus*	57	2	55	1 (2)	53	54
*V. anguillarum*	4	1	3	0	3	3
*V. cholerae*	177	14	163	29 (2–10)	68	97
*V. diabolicus*	57	5	52	3 (2)	46	49
*V. harveyi*	1	0	1	0	1	1
*V. metschnikovii*	10	4	6	1 (6)	0	1
*V. parahaemolyticus*	179	18	161	30 (2–8)	73	103
*V. ziniensis*	4	1	3	1 (3)	0	1
Total	489	45	444	65 (2–10)	244	309

^*a*^ Pairwise analysis of genome similarity was used to identify duplicate genomes when two or more sequenced isolates arose from the same sample or host. After removal of duplicate genomes, clusters of near-identical or identical genomes from different samples and hosts were still common, especially in *V. cholerae* and *V. parahaemolyticus*. Genomes that were not closely related to any others were considered singletons. The total unique genomes were composed of the sum of singletons and the number of genome clusters.

Genome clusters were rare in *V. alginolyticus* and *V. diabolicus* and occurred in genome pairs sampled the same day up to 2 months apart (Table 1). Clusters of similar genomes were common in *V. cholerae* and *V. parahaemolyticus*, and many arose from spatiotemporally localized sampling events of environmental samples, southern sea otter feces, or avian feces. Ten out of 11 clusters with five or more genomes contained at least two genomes collected from the same location within a month, with four clusters composed of only environmental strains from the same sampling event (Table 2). Seven of the 11 clusters contained at least one genome from disparate sampling efforts with five clusters from mixed isolation sources. One cluster of eight *V*. *parahaemolyticus* genomes ranged from Moss Landing, CA, to Cayucos, CA, and included samples from shellfish (2002), southern sea otters (2002–2006), and the environment (2019).

**TABLE 2:** Table collection details on genome clusters comprised of five or more genomes from the broader *Vibrio* species sampling efforts in Alaska, California, and Washington from 2000 to 2019, including the isolation sources and notes on localized genome sharing^*a*^

Cluster size	Species	BCW sample IDs	Sources	Collection notes
10	*V. cholerae*	11447, 11449, 11451, 11457, 11463, 11464, 11424, 11427, 11429, 11440	10 environment	Localized collection May–June 2010 near Andrew Molera SP, CA.
8	*V. cholerae*	11175, 12267, 11448, 11459, 11425, 11432, 11433, 11435	1 avian, 7 environment	Environment-localized collection May–June 2010 near Andrew Molera SP, CA. Avian feces collected in 2007 from a San Francisco Bay wildlife hospital.
8	*V. parahaemolyticus*	12103, 12176, 12180, 12209, 12276, 12148, 11036, 11076	2 environment, 2 shellfish, 4 SSO	Environment-localized collection June 2019 in Elkhorn Slough, near Moss Landing, CA. Shellfish collected in 2002 from Monterey Bay. SSO collected 2003–2006 from Monterey, CA to Cayucos, CA.
6	*V. metschnikovii*	11063, 11066, 11080, 11082, 11085, 11087	6 environment	Localized collection June 2019 in Elkhorn Slough, near Moss Landing, CA.
6	*V. parahaemolyticus*	11034, 11074, 11166, 11543, 12166, 12167	1 environment, 5 SSO	Environment collected June 2019 in Elkhorn Slough, near Moss Landing, CA. SSO sampled in 2008 (4) and 2012 between Moss Landing, CA and Lucia, CA.
5	*V. cholerae*	11098, 11100, 11188, 11189, 12147	5 SSO	Localized collection of 4 otters Feb. 2008 near Moss Landing, CA. One additional collected in 2006 near Piedras Blancas, CA.
5	*V. cholerae*	11117, 11485, 12246, 12256, 12258	4 avian, 1 SSO	Localized collection of 3 avian feces Nov. 2007 from a San Francisco Bay wildlife hospital. One avian feces sampled near Carmel, CA in Aug. 2007. SSO sampled Oct. 2007 near Moss Landing, CA. All isolates from 2007.
5	*V. cholerae*	11141, 11157, 11143, 11483, 11484	3 avian, 2 SSO	Localized collection of avian feces Aug.–Sep. 2007 near Carmel, CA. SSO collected in 2008 near Moss Landing, CA.
5	*V. cholerae*	11178, 11442, 11444, 11426, 11434	5 environment	Localized collection May–June 2010 near Andrew Molera SP, CA.
5	*V. cholerae*	11443, 11445, 11454, 11456, 11458	5 environment	Localized collection May–June 2010 near Andrew Molera SP, CA.
5	*V. parahaemolyticus*	12127, 12305, 12325, 12326, 12327	5 SSO	Localized collection of 2 SSO Nov.–Dec. 2005 near Morro Bay, CA. Remaining 3 collected from 2004 to 2006 ranging from near Pajaro Dunes, CA to near Vandenberg Air Force Base, CA.

^*a*^ Sources labeled SSO are isolated from southern sea otters.

### Antimicrobial resistance phenotypes in southern sea otters

**Fig 2:**
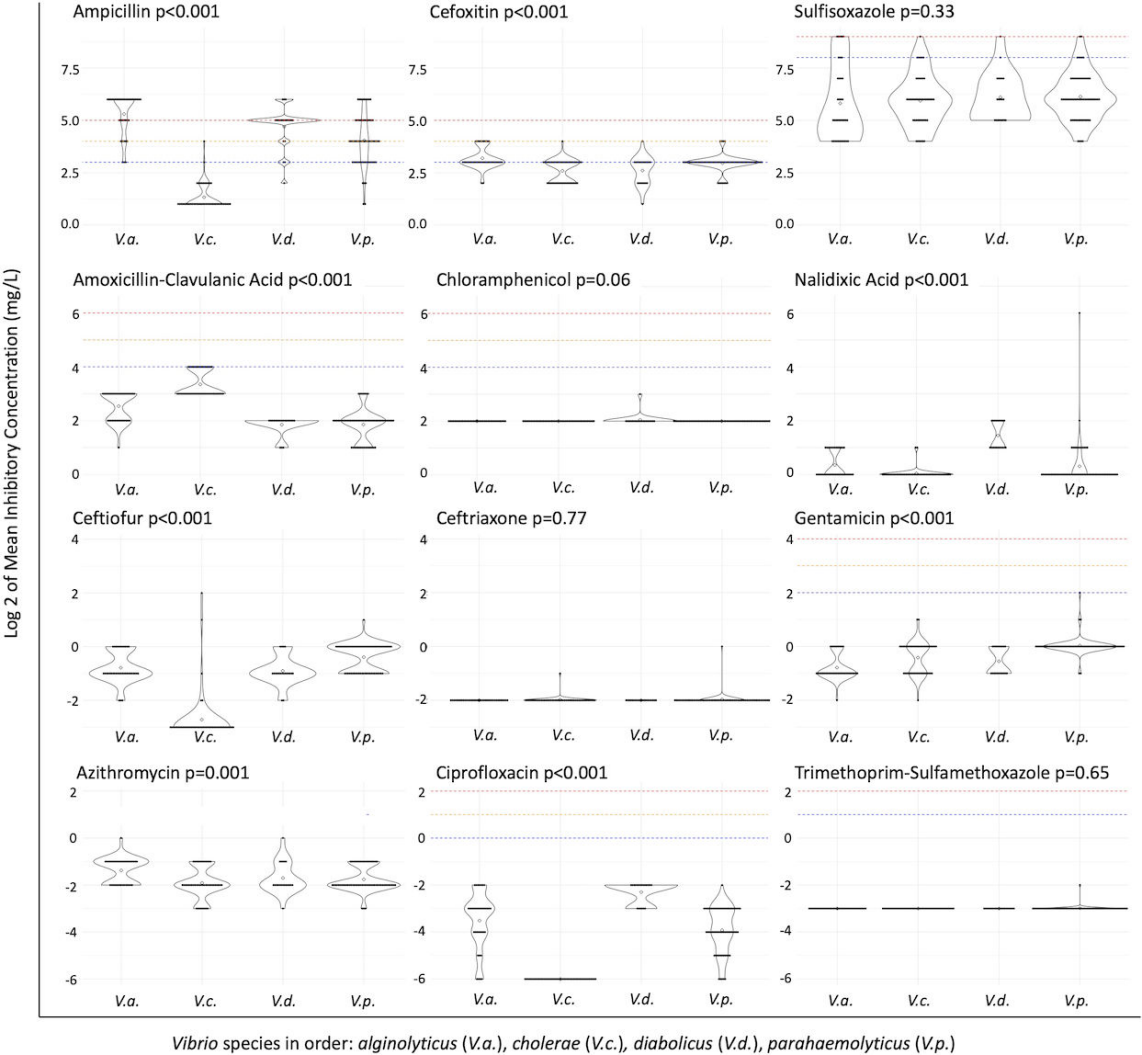
Violin plot of antimicrobial susceptibility testing to 13 antibiotics by microbroth dilution of a subset of *n* = 37 *V*. *alginolyticus*, *n* = 46 *V*. *cholerae*, *n* = 20 *V*. *diabolicus*, and *n* = 62 *V*. *parahaemolyticus* isolated from southern sea otters off the California coast between 2000 and 2013. *Vibrio* species differed in their minimum inhibitory concentration to eight different antibiotics based on Kruskal-Wallis *P* values less than 0.05. All isolates exhibited a minimum inhibitory concentration to tetracycline of ≤4 mg/L (not pictured). Dashed lines represent the clinical laboratory standards institute’s breakpoints for susceptible (blue), intermediate (orange), and resistant (red) interpretations for each antibiotic where available. Sulfisoxazole breakpoints are validated for *V. cholerae* only.

The expected MIC of eight tested antibiotics differed between *Vibrio* spp., although resistance was only observed for ampicillin and sulfisoxazole; intermediate interpretations were also observed for cefoxitin (Fig. 2). Ampicillin was the most frequently observed resistance; *V. alginolyticus* [78.4%, 95% confidence interval (CI): 62.8, 88.6] and *V. diabolicus* (75%, 95% CI: 53.1, 88.8) were 7.1 (95% CI: 2.8, 18.2, *P* < 0.001) and 5.9 (95% CI: 1.9, 18.3, *P* = 0.002) times more likely, respectively, to be resistant to ampicillin than *V. parahaemolyticus* (33.9%, 95% CI: 23.3, 46.3), while only one *V. cholerae* isolate was intermediate to ampicillin. Cefoxitin intermediate isolates were 4.7 (95% CI: 1.3–16.5, *P* = 0.02) times more common in *V. alginolyticus* (24.3%, 95% CI: 13.4, 40.1) than *V. parahaemolyticus* (6.5%, 95% CI: 2.5, 15.4) and were also uncommon in *V. diabolicus* (5.0%, 95% CI: 0.9, 23.6) and *V. cholerae* (2.2%, 95% CI: 0.4, 11.3). Only nine isolates were sulfisoxazole resistant (5 *V*. *alginolyticus*, 1 *V*. *cholerae*, 1 *V*. *diabolicus*, and 2 *V*. *parahaemolyticus*). *Vibrio* spp. phenotypes of AMR were not associated with southern sea otter age class or sex, nor were there spatial clusters observed.

No multidrug-resistant isolates were detected based on a criterion of phenotypic resistance to three or more drug classes. However, the sulfisoxazole-resistant *V. alginolyticus*, *V. diabolicus*, and *V. parahaemolyticus* isolates were also resistant to ampicillin, with one *V*. *parahaemolyticus* isolate also interpreted as intermediate to cefoxitin. Only one northern sea otter isolate (*V. cholerae*) had antimicrobial susceptibility testing results, and it was susceptible to amoxicillin-clavulanic acid, ampicillin, ceftiofur, gentamicin, tetracycline, and trimethoprim-sulfamethoxazole.

### Comparison of genotypic and phenotypic resistance

Antimicrobial resistance genes detected in *Vibrio* spp. putatively confer resistance to multiple drug classes including ß-lactams, tetracyclines, sulfonamides, fluoroquinolones, and chloramphenicol (Tables 3 and 4). Genes associated with multidrug efflux pumps and resistance to ß-lactams and tetracyclines were frequently detected while genes associated with resistance to fluoroquinolones, aminoglycosides, chloramphenicol, and sulfonamides were uncommon. However, the frequencies of gene detections were not reliably associated with phenotypic antibiotic resistance.

**TABLE 3:** Prevalence of antimicrobial resistance genes in genomes from *Vibrio alginolyticus* and *Vibrio diabolicus* collected in Alaska, California, and Washington from 2000 to 2019 separated by isolation sources^*a*^

		*V. alginolyticus* count (%)						*V. diabolicus* count (%)					
Drug class	Gene	Avian (*n* = 0)	Environ (*n* = 7)	NSO (*n* = 6)	Shell (*n* = 3)	SSO (*n* = 39)	Total (*n* = 55)	Avian (*n* = 1)	Environ (*n* = 14)	NSO (*n* = 5)	Shell (*n* = 12)	SSO (*n* = 20)	Total (*n* = 52)
Aminoglycoside	*aph(3")-Ib*												
	*aph(6)-Id*												
β-lactam	*varG*												
	*bla*CARB-7												
	*bla*CARB-42		7 (100)	6 (100)	3 (100)	39 (100)	55 (100)	1 (100)	14 (100)	5 (100)	12 (100)	20 (100)	52 (100)
	*bla*CARB-18 *to bla*CARB-46												
	*ampC*		5 (71.4)	5 (83.3)	3 (100)	28 (71.8)	41 (74.5)	1 (100)	9 (64.3)	5 (100)	7 (58.3)	19 (95)	41 (78.8)
	*ACT/blaCMY-2*												
Diaminopyrimidine	*dfrA1*												
	*dfrA6*												
Fluoroquinolone	*qnrS5*					1 (2.6)	1 (1.8)						
	*qnrVC4*												
	*qnrVC6*								1 (7.1)				1 (1.9)
	*qnrVC10*					1 (2.6)	1 (1.8)						
Fosfomycin	*fosA*					2 (5.1)	2 (3.6)		5 (35.7)	1 (20)	5 (41.7)	9 (45)	20 (38.5)
Multidrug efflux	*CRP*		7 (100)	6 (100)	3 (100)	39 (100)	55 (100)	1 (100)	14 (100)	5 (100)	12 (100)	20 (100)	52 (100)
	*EMRD*												
	*vceCAB, vceR*												
	*vcmA*		7 (100)	6 (100)	3 (100)	39 (100)	55 (100)	1 (100)	14 (100)	5 (100)	12 (100)	20 (100)	52 (100)
	*vexAB*		7 (100)	6 (100)	3 (100)	39 (100)	55 (100)	1 (100)	14 (100)	5 (100)	12 (100)	20 (100)	52 (100)
	*vexC*												
	*vexD*												
	*vexE*												
	*vexF*		7 (100)	6 (100)	3 (100)	39 (100)	55 (100)	1 (100)	14 (100)	5 (100)	12 (100)	20 (100)	52 (100)
	*vexH*		7 (100)	6 (100)	3 (100)	39 (100)	55 (100)	1 (100)	14 (100)	5 (100)	12 (100)	20 (100)	52 (100)
	*vexK*		7 (100)	6 (100)	3 (100)	39 (100)	55 (100)	1 (100)	14 (100)	5 (100)	12 (100)	20 (100)	52 (100)
Peptide (polymyxin)	*almG*												
	*UGD*		5 (71.4)	5 (83.3)	1 (33.3)	18 (46.2)	29 (52.7)		8 (57.1)	1 (20)	5 (41.7)	12 (60)	26 (50)
Phenicol	*catB9*												
	*floR*												
Sulfonamide	*sul1*												
	*sul2*												
Tetracycline	*tet34*		7 (100)	6 (100)	3 (100)	39 (100)	55 (100)	1 (100)	14 (100)	5 (100)	12 (100)	20 (100)	52 (100)
	*tet35*		7 (100)	6 (100)	3 (100)	39 (100)	55 (100)	1 (100)	14 (100)	5 (100)	12 (100)	20 (100)	52 (100)
	*tetA*												

^*a*^ Not described in the table are *V. anguillarum* (*n* = 3), *V. harveyi* (*n* = 1), *V. metschnikovii* (*n* = 6), and *V. ziniensis* (*n* = 3), which all carried *tet34*, *tet35*, *CRP, vexA*, *vexB*, and *vexH*. In addition, the *V. anguillarum* genomes carried *vcmA*, *vexF*, and *vexK* (33%); *V. harveyi* carried *bla*_VHH-1_, *vcmA*, *vexF*, and *vexK; V. metschnikovii* carried *vcmA*, *bla*_CARB-4_, and *ugd*; and *V. ziniensis* carried *catB9*, *vceA*, *vceB*, *vceR*, and *vexF*. Isolation sources include avian feces (Avian), environmental sampling (Environ), northern sea otters (NSO), shellfish (Shell), and southern sea otters (SSO).

**TABLE 4:** Prevalence of antimicrobial resistance genes in genomes from *Vibrio cholerae* and *Vibrio parahaemolyticus* collected in Alaska, California, and Washington from 2000 to 2019 separated by isolation sources^*a*^

		*V. cholerae* count (%)						*V. parahaemolyticus* count (%)					
Drug class	Gene	Avian (*n* = 50)	Environ (*n* = 52)	NSO (*n* = 5)	Shell (*n* = 12)	SSO (*n* = 44)	Total (*n* = 163)	Avian (*n* = 1)	Environ (*n* = 29)	NSO (*n* = 21)	Shell (*n* = 4)	SSO (*n* = 106)	Total (*n* = 161)
Aminoglycoside	*aph(3")-Ib*										1 (25)		1 (0.6)
	*aph(6)-Id*										1 (25)		1 (0.6)
β-lactam	*varG*	24 (48)	14 (26.9)	2 (40)	10 (83.3)	23 (52.3)	73 (44.8)						
	*bla*CARB-7	5 (10)	14 (26.9)				19 (11.7)						
	*bla*CARB-42												
	*bla*CARB-18 to *bla*CARB-46							1 (100)	29 (100)	21 (100)	4 (100)	106 (100)	161 (100)
	*ampC*												
	*ACT/blaCMY-2*										1 (25)		1 (0.6)
Diaminopyrimidine	*dfrA1*										1 (25)		1 (0.6)
	*dfrA6*									1 (4.8)			1 (0.6)
Fluoroquinolone	*qnrS5*												
	*qnrVC4*	5 (10)				1 (2.3)	6 (3.7)						
	*qnrVC6*												
	*qnrVC10*												
Fosfomycin	*fosA*				1 (8.3)	2 (4.5)	3 (1.8)					2 (1.9)	2 (1.2)
Multidrug efflux	*CRP*	50 (100)	52 (100)	5 (100)	12 (100)	44 (100)	163 (100)	1 (100)	29 (100)	21 (100)	4 (100)	106 (100)	161 (100)
	*emrD*	3 (6)	7 (13.5)	2 (40)	4 (33.3)	6 (13.6)	22 (13.5)						
	*vceCAB, vceR*	23 (46)	34 (65.4)	5 (100)	5 (41.7)	26 (59.1)	93 (57.1)						
	*vcmA*	50 (100)	52 (100)	5 (100)	12 (100)	44 (100)	163 (100)	1 (100)	29 (100)	21 (100)	4 (100)	106 (100)	161 (100)
	*vexAB*	50 (100)	52 (100)	5 (100)	12 (100)	44 (100)	163 (100)	1 (100)	29 (100)	21 (100)	4 (100)	106 (100)	161 (100)
	*vexC*	50 (100)	52 (100)	5 (100)	12 (100)	44 (100)	163 (100)						
	*vexD*	50 (100)	52 (100)	5 (100)	12 (100)	44 (100)	163 (100)			1 (4.8)		12 (11.3)	13 (8)
	*vexE*	50 (100)	52 (100)	5 (100)	12 (100)	44 (100)	163 (100)						
	*vexF*	50 (100)	52 (100)	5 (100)	12 (100)	44 (100)	163 (100)	1 (100)	29 (100)	21 (100)	4 (100)	106 (100)	161 (100)
	*vexH*	50 (100)	52 (100)	5 (100)	12 (100)	44 (100)	163 (100)	1 (100)	29 (100)	21 (100)	4 (100)	106 (100)	161 (100)
	*vexK*	50 (100)	50 (96.2)	5 (100)	12 (100)	44 (100)	161 (98.8)	1 (100)	29 (100)	21 (100)	4 (100)	106 (100)	161 (100)
Peptide (polymyxin)	*almG*	49 (98)	49 (94.2)	5 (100)	11 (91.7)	44 (100)	158 (96.9)						
	*UGD*	8 (16)			2 (16.7)	3 (6.8)	13 (8)	1 (100)	8 (27.6)	3 (14.3)	3 (75)	39 (36.8)	54 (33.5)
Phenicol	*catB9*	3 (6)	10 (19.2)	2 (40)		3 (6.8)	18 (11)						
	*floR*										1 (25)		1 (0.6)
Sulfonamide	*sul1*										1 (25)		1 (0.6)
	*sul2*										1 (25)		1 (0.6)
Tetracycline	*tet34*	50 (100)	52 (100)	5 (100)	12 (100)	44 (100)	163 (100)	1 (100)	29 (100)	21 (100)	4 (100)	106 (100)	161 (100)
	*tet35*	50 (100)	52 (100)	5 (100)	12 (100)	44 (100)	163 (100)	1 (100)	29 (100)	21 (100)	4 (100)	106 (100)	161 (100)
	*tetA*										1 (25)		1 (0.6)

^*a*^ Isolation sources include avian feces (Avian), environmental sampling (Environ), northern sea otters (NSO), shellfish (Shell), and southern sea otters (SSO).

Tetracycline resistance genes *tet34* and *tet35* were ubiquitous in all *Vibrio* species, although southern sea otter isolates with antimicrobial susceptibility testing (*n* = 62) were susceptible to tetracycline. All but one *tet34* gene had a percent coverage of at least 98.9%, but the percent identity was between 75.1% and 83.9%. For *tet35* genes, the percent coverage ranged between 70.8% and 100%, and the percent identity ranged between 71.7% and 99.4%. A similar discordance was observed between the sulfisoxazole resistance phenotype and genotype; sulfonamide drug class resistance genes *sul1* and *sul2* were detected in a single *V. parahaemolyticus* isolate from shellfish and was not detected in the five sulfisoxazole-resistant southern sea otter isolates. The *sul1-* and *sul2*-positive shellfish isolate was also the only isolate to carry the following genes: *tetA*, *blaCMY-2*/*act*, *aph3-lb*, *aph6-ld*, *dfrA1*, and *floR*.

Fluoroquinolone resistance *qnr* genes were detected in only 2 *V. alginolyticus* genomes, 1 *V. diabolicus* genome, and 6 *V. cholerae* genomes. The *fosA* gene, which confers fosfomycin resistance, was rare in *Vibrio* spp. except in *V. diabolicus* (40.4%, 95% CI: 27.3, 54.9). The chloramphenicol resistance gene *catB9* was only detected in *V. cholerae* (11%, 95% CI: 6.9, 17.1), although all tested *V. cholerae* isolates were susceptible to chloramphenicol. The peptide resistance gene *almG* was nearly ubiquitous in *V. cholerae* but absent from the other three *Vibrio* species. Various multiple drug efflux pumps, including the nearly ubiquitous *vexAB*, were detected but were not associated with phenotypic resistance patterns.

At least one gene that putatively confers ß-lactam resistance was present in all species included in this study except *V. anguillarum* and *V. ziniensis*, but species differed in the specific resistance gene that was present. The genes *varG* and *bla*_CARB-7_ were only detected in *V. cholerae* with the *bla*_CARB-7_ gene detected in 11.7% of *V. cholerae* isolates (95% CI: 7.3, 17.8) but only those from avian feces (10%) and environmental sources (26.9%). The gene *varG* was detected in 44.7% (95% CI: 37.1, 52.8) of *V. cholerae* isolates, but the presence of *varG* was not associated with the MIC of amoxicillin/clavulanic acid or ampicillin. The *bla*_CARB-42_ gene was ubiquitous in *V. alginolyticus* and *V. diabolicus* isolates, although a blast of the protein-coding sequence from *V. diabolicus* genomes most closely matched a recently discovered allelic variant *bla*_CARB-57_ that was not yet added to the reference databases (70). The *ampC* gene, which putatively confers resistance to cephalosporins, was detected in 74.5% (95% CI: 60.7, 84.9) of *V. alginolyticus* and 78.8% (95% CI: 64.9, 88.5) of *V. diabolicus*, but the presence of *ampC* was not associated with an increased MIC to ß-lactams.

**Fig 3:**
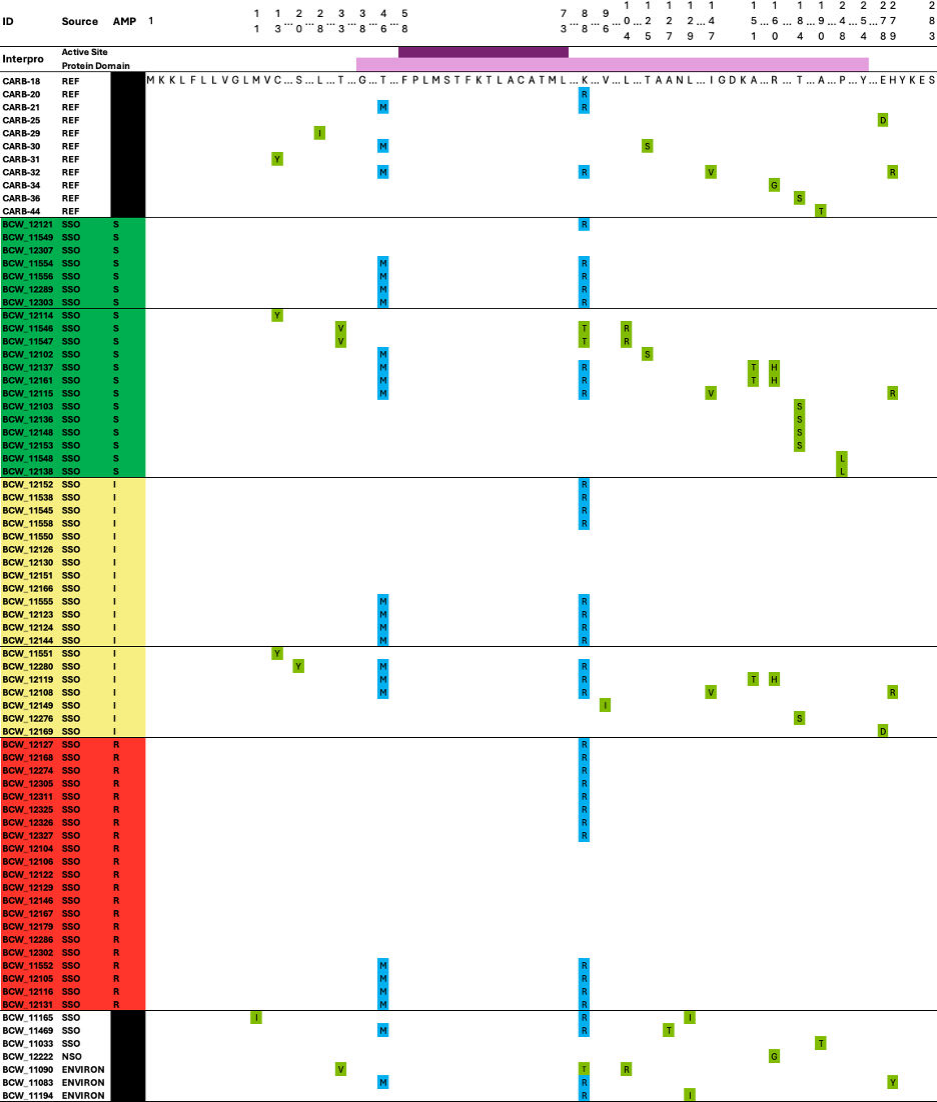
Amino acid sequence alignment of translated *bla*_CARB_ gene sequences from 61 *Vibrio parahaemolyticus* isolates from southern sea otters in California (2000–2013) separated by ampicillin (AMP) susceptibility testing interpretation and compared to 11 reference CARB protein sequences (top rows) from the Comprehensive Antibiotic Resistance Database. All sequences from ampicillin-resistant isolates aligned exactly with proteins CARB-18, CARB-20, or CARB-21. Sequences with at least one additional amino acid substitution were more likely to be susceptible to ampicillin than sequences that matched CARB-18, CARB-20, or CARB-21 proteins (OR = 9.0 [2.6, 30.8], *P* < 0.001). Isolate IDs on the left column are color coded based on antibiotic susceptibility testing as ampicillin susceptible (S – green, *n* = 20), intermediate (I – yellow, *n* = 20), or resistant (R – red, *n* = 21). Seven additional isolates from environmental samples (ENVIRON), a northern sea otter (NSO), and southern sea otters (SSO) collected between 2008 and 2019 are included (white) to represent the additional variability in sequences observed across sequenced *V. parahaemolyticus* isolates. Amino acid substitutions T46M and K88R, which differentiate CARB-18, CARB-20, and CARB-21, are highlighted in blue, while all other amino acid substitutions are highlighted in light green. Ellipses denote abridged sections of the protein sequence. Interpro classification was utilized to predict the protein domain (light purple line above sequences; residues 38–254) and the active site (dark purple; residues 58–73).

Allelic variants of the *bla*_CARB_ gene were present in all *V. parahaemolyticus* genomes. Either *bla*_CARB-18_, *bla*_CARB-20_, or *bla*_CARB-21_, which differs only at amino acid residues 46 and 88 in their protein-coding region, was present in all ampicillin-resistant isolates (Fig. 3). The presence of additional amino acid substitutions other than AA46 and AA88 in the protein-coding region occurred in 32.8% (95% CI: 21.6, 46.1) of the *V. parahaemolyticus* isolates with antimicrobial susceptibility testing. Isolates with additional amino acid substitutions were 9.02 times (95% CI: 2.6, 30.8) more likely to be susceptible to ampicillin than those with *bla*_CARB-18_, *bla*_CARB-20_, or *bla*_CARB-21_ alleles (*x^2^* = 14.0 *, P* < 0.001). The predicted active site of the CARB protein (AA58-73) was conserved across all *V. parahaemolyticus* genomes, although the predicted protein domain (AA38-254) included the majority of CARB protein substitutions. Of the 13 susceptible *V. parahaemolyticus* isolates with CARB amino acid substitutions, 12 had at least one substitution within the predicted protein domain.

The presence of additional substitutions did not differ between wet (December–May) and dry seasons (June–November), male and female otters, adults and juvenile/subadult otters, live versus dead sampling, and spatial distribution. Additional substitutions in the translated *bla*_CARB_ sequence did not significantly differ between environmental isolates and southern sea otters but were 3.4 times (95% CI: 1.3, 9.1) more common in northern sea otter isolates compared to southern sea otters (*P* = 0.009).

Clusters of near-identical *V. parahaemolyticus* also shared identical *bla*_CARB_ genotypes but not always the exact same ampicillin MIC during antimicrobial susceptibility testing. The cluster of eight *V. parahaemolyticus* (Table 2) included three isolates with susceptibility testing results, including one ampicillin intermediate and two ampicillin-susceptible isolates. These isolates all featured an amino acid substitution (T184S) within the predicted protein domain. The cluster of six *V. parahaemolyticus* strains included an ampicillin-resistant and an ampicillin intermediate strain, and featured a protein matching CARB-18. Meanwhile, all clusters of five *V. parahaemolyticus* strains were ampicillin resistant and featured a protein matching CARB-20. Near-identical isolates always shared the same predicted CARB protein, and clusters did not contain both susceptible and resistant strains, despite overlap in susceptible and intermediate, or intermediate and resistant classification.

## DISCUSSION

### Species identification and genomic similarity

Because pathogenic *Vibrio* species are often closely related to more benign species, accurate species identification is crucial to assess health threats to humans, seafood, and marine wildlife (71, 72). By utilizing genomic-based species identification, we differentiated 52 *V. diabolicus* isolates (49 unique genome types) that were previously identified as *V. alginolyticus* or *V. parahaemolyticus. Vibrio diabolicus* is most closely related to *V. alginolyticus*, has been detected in diverse sources and origins, and due to its recent discovery, has limited numbers of sequenced genomes (2, 3, 73). The additional *V. diabolicus* genomes in this data set represent the largest publicly available set of *V. diabolicus* genomes and include the first confirmation of northern and southern sea otter infection. Prior misclassification of *V. diabolicus* and *V. alginolyticus* may have important implications for the interpretation of earlier research regarding AMR and virulence of both species.

Clinical typing methods, including MALDI-ToF, have relatively low species resolution due to insufficiently populated *Vibrio* spp. databases, and thus may mischaracterize understudied and emerging pathogens (74). Biochemical testing and MALDI-ToF identification methods are not currently optimized for *V. diabolicus* detection due to limited data (75). To improve the resolution of MALDI-ToF for *V. diabolicus*, a custom set of reference mass spectra profiles informed by genomics to represent species diversity could be added; a custom database approach has been effective to improve species identification in other Harveyi clade species (74).

Species classification and sequence typing by MLSA/MLST methods may also result in inadequate resolution of *V. diabolicus*, as was observed using the PubMLST MLSA and MLST schemes. While other multilocus schemes may prove superior to the current PubMLST schemes (75–77), multilocus schemes still do not take advantage of all available sequencing data provided the same way that whole genome-based classifications can. Improved diagnostics for *V. diabolicus* identification will allow for future studies to investigate the pathogenicity and epidemiology in human and marine hosts.

In addition to accurate species detection, genome distance comparisons indicated both high inter- and intra-species genome diversity, as expected for *Vibrio* species (7, 73, 78, 79). The pairwise genomic comparisons also identified clusters of near-identical or identical genomes, which can be used to identify potential selection bias, generate hypotheses around circulation of *Vibrio* spp. strains within localized populations, as well as more widespread transmission patterns. Intensive sampling events included feces from multiple sea otters or multiple gulls (*Larus* spp.) from the same beach, or collection of environmental samples from multiple locations within the same watershed (8, 9). These events were characterized by multiple nearly-identical or identical genomes, which may suggest localized circulation of specific strains. Certain strains of *V. parahaemolyticus* have been detected within specific isolation sources and may be localized in outbreak scenarios (7, 80).

Some genome clusters were instead composed of isolates from mixed sources and indicate potential for cross-species transmission, with marine wildlife potentially acting as reservoirs. Aquatic birds have been proposed as potential reservoirs and vectors for pathogenic *Vibrio* spp. (81–83); sea otters and other marine mammals could represent additional reservoirs that should be further investigated and sequenced. Other genome clusters were composed of isolates from disparate space-time points indicative of long-distance transmission and environmental persistence. Natural processes such as ocean currents or human activities such as shipping provide opportunities to disseminate highly related strains to geographically distant locations (84, 85). Genomic stability of phylogenomic *V. parahaemolyticus* clusters detected over 10-plus years suggests that (i) at least some *Vibrio* lineages can be extremely stable over time, including their non-core genes, and (ii) related genomes may arise from persistent but undetected reservoirs that can appear sporadically in different times and locations (80, 86).

### Antimicrobial susceptibility in sea otters

The subset of southern sea otter isolates tested for antimicrobial susceptibility was susceptible to most clinically appropriate drugs tested. Ampicillin resistance was the most common drug resistance observed in *V. alginolyticus*, *V. diabolicus*, and *V. parahaemolyticus*, although all tested *V. cholerae* isolates were susceptible to ampicillin. Few *Vibrio* spp. isolates were characterized as cefoxitin intermediate. Ampicillin MICs were never higher than 16 µg/mL (intermediate) in the only previous southern sea otter antimicrobial susceptibility study (8). Previous *V. parahaemolyticus* studies in the United States reported approximately 50% prevalence of ß-lactam drug resistance or higher, while globally, some studies reported as high as 100% prevalence (5, 22, 24, 25). Few studies have reported antimicrobial susceptibility testing for whole genome-sequenced *V. alginolyticus* and *V. diabolicus* (listed as formerly separate species *V. antiquarius*), but in those studies, both species exhibited a high prevalence of ß-lactam drug resistance and *bla*_CARB_ gene detection (29, 87). Prevalence of ß-lactam-resistant *V. cholerae* appears to be less frequent than the other three species, which corresponds with the lack of *bla*_CARB-7_-positive and ß-lactam-resistant *V. cholerae* southern sea otter isolates in our study (88–90).

Presumptive sulfisoxazole resistance was observed infrequently in all four *Vibrio* species, but the breakpoint for sulfisoxazole (512 µg/mL) is currently only valid for *V. cholerae* (67). A breakpoint for sulfisoxazole of 256 µg/mL could be useful given the distribution of MIC values and the lack of breakpoints for all *Vibrio* species, especially those intended to inform treatment of stranded marine wildlife. While we did not find evidence of multiple drug resistance or resistance to additional drug classes beyond ß-lactams and sulfonamides, resistance to higher-class cephalosporins, chloramphenicol, erythromycin, fluoroquinolones, tetracyclines, or trimethoprim is a potential clinical concern, as resistance to each of these drugs has been previously documented (25, 91, 92).

Human clinical cases of vibriosis in the United States are treated with quinolones (56.1%), cephalosporins (24.1%), tetracyclines (23.5%), and penicillins (15.4%), although penicillins alone are not typically effective (93). Our data suggest the antibiotics used in human infections (quinolones, cephalosporins, and tetracycline) are currently good choices to treat vibriosis in stranded sea otters, but AMR patterns should be monitored routinely.

### Discordance between AMR genotype and phenotype

Straightforward associations between AMR genotypes and phenotypes are documented for some highly characterized microbial pathogens with known mechanisms of resistance, thorough gene databases, and validated antimicrobial susceptibility test interpretations. Previous studies that were able to accurately predict AMR phenotypes using whole genome sequencing with gene databases utilized bacterial species with known mechanisms of resistance and well-validated antimicrobial susceptibility test interpretation (62, 94, 95). Genotype–phenotype comparison is complicated in *Vibrio* spp. by extrapolated phenotypic interpretations, incomplete databases for *Vibrio*-specific genes, and gaps in knowledge about the genetic mechanisms of resistance, including factors impacting variable gene expression (29).

Genotype–phenotype comparison was examined for ampicillin resistance. Comparison of the *bla*_CARB-7_ genotype and ampicillin resistance phenotype for *V. cholerae* could not be performed because no sea otter isolates carried *bla*_CARB-7_. Previously, 57% of non-O1/O139 *V. cholerae* isolates characterized as ampicillin intermediate or resistant harbored *bla*_CARB-7_, suggestive of a partial association between genotype and phenotype (88). All *V. parahaemolyticus*, *V. alginolyticus*, and *V. diabolicus* genomes in the current study possessed a copy of a *bla*_CARB_ family gene, but despite their ubiquity, only 78.4%, 76.2%, and 33.9% of *V. alginolyticus*, *V. diabolicus*, and *V. parahaemolyticus* isolates, respectively, were ampicillin resistant. In-depth examination of the predicted CARB protein sequence from *V. parahaemolyticus* genomes further elucidates the discordance between *bla*_CARB_ presence and ampicillin susceptibility.

The *bla*_CARB-17_ gene family was only recently discovered and was thought to be intrinsic to the *V. parahaemolyticus* genome due to its position in the genome and ubiquitous detection in GenBank accessions (96). Nomenclature for AMR genes is inconsistent in literature and across AMR databases, and includes allelic variants that can vary by as few as one or two amino acids. In this study, 17 different *bla*_CARB_ genes were detected for *V. parahaemolyticus*, each with different amino acid substitutions in the protein-coding region that may alter gene function or antibiotic specificity.

Alignment of the CARB proteins revealed that rare amino acid substitutions partially explain phenotypic ampicillin resistance. Some of the rare amino acid substitutions observed appear to be conservative, and most of the substitutions occurred within the predicted protein domain. While not all the substitutions are likely to have an equal effect, the impact of one or more substitutions on enzyme activity or secretion of CARB proteins is worthy of further investigation. Sequences that had a 100% match to the protein sequences of *bla*_CARB-18_, *bla*_CARB-20_, or *bla*_CARB-21_ were more likely to be ampicillin resistant. Structural analysis of *bla*_CARB-20_ confirmed a preference for non-cephalosporin ß-lactams like ampicillin; this plasticity in amino acid substitutions should continue to be monitored for potential functional alterations such as reduced or increased drug binding (97). Discordance between antimicrobial-resistant phenotypes and genotypes arise from multiple factors, including imprecise phenotypic methodology and differential gene expression. The two-component system *vbrK*/*vbrR* regulates the expression of *bla*_CARB_ genes and type III secretion system genes, which results in greater *bla*_CARB_ gene expression in the presence of ß-lactams (98–100). Future investigations should consider these additional physiological and genetic complexities when interpreting the presence or absence of various AMR genes.

Unexplained discord was noted in the genotype and phenotype for other antimicrobial drug classes including sulfonamides and tetracyclines. The discordance between the rarity of *sul1* and *sul2* sulfonamide resistance genes and the variation in MICs suggests the presence of other genetic mechanisms that *Vibrio* spp. can harbor to deal with sulfonamide class drugs, including mechanisms to transform sulfonamides through metabolic pathways (101). Conversely, tetracycline resistance genes *tet34* and *tet35* were ubiquitous while isolates were phenotypically susceptible. The *tet34* gene is dependent on MgCl_2_, so variable MgCl_2_ concentrations could impact antibiotic susceptibility test results (102). While *tet35* works as an energy-dependent efflux pump, it may be less effective than other tetracycline efflux pumps such as *tetA* (103). Additionally, the percent identities of *tet34* and *tet35* in the study genomes were relatively low compared to the reference sequence (70%–90%), which may suggest a less functional or non-functional allelic variant.

### Genomic epidemiology and antimicrobial resistance

Many of the AMR genes identified were ubiquitous within host species and distributed across *Vibrio* spp. However, the genomic epidemiology approach utilized identified potential for niche-adapted ß-lactam resistance profiles. For example, the *bla*_CARB-7_ genotype of *V. cholerae* was not detected in northern or southern sea otter isolates in the current study but was detected in avian and environmental *V. cholerae* isolates. For *V. parahaemolyticus*, substitutions to the *bla*_CARB_ genes of *V. parahaemolyticus* were more frequently observed in northern sea otters than in southern sea otters or California environmental isolates. These two examples highlight how isolation sources can impact observations into AMR adaptations in *Vibrio* spp.

Clusters of near-identical *V. parahaemolyticus* also shared identical *bla*_CARB_ genotypes but not always the exact same ampicillin MIC during antimicrobial susceptibility testing. The cluster of eight *V. parahaemolyticus* (Table 2) included three isolates with susceptibility testing results, including one ampicillin intermediate and two ampicillin-susceptible isolates. These isolates all featured an amino acid substitution (T184S) within the predicted protein domain. The cluster of six *V. parahaemolyticus* strains included an ampicillin-resistant and an ampicillin intermediate strain, and featured a protein matching CARB-18. Meanwhile, the cluster of five *V. parahaemolyticus* strains were all ampicillin resistant and featured a protein matching CARB-20. Near-identical isolates always shared the same predicted CARB protein, and clusters did not contain both susceptible and resistant strains, despite overlap in susceptible and intermediate, or intermediate and resistant classification.

The clusters of near-identical *Vibrio* spp. strains also provide important AMR insights that could be beneficial to environmental and public health monitoring. Results of multiple *V. parahaemolyticus* clusters with both genomic and phenotypic AMR data suggest that stable genome strains capable of temporal persistence, localized circulation, and/or long-distance transmission can also maintain consistent antimicrobial-resistant genotypes and phenotypes. Therefore, continued monitoring could help detect recurrence of antimicrobial-resistant strains.

### Limitations and future directions

The interpretation of the statistical methods used relies on the assumption of independence between observations. Selection bias during sampling may result in some level of dependence between isolates sampled in spatiotemporal proximity. Genomic relatedness analysis helped reduce this bias through the detection and removal of nearly-identical genomes that were sampled from the same host or population. Future studies can utilize a genomic epidemiology approach to further investigate the role of marine mammal hosts, such as sea otters, as bioindicators of antimicrobial-resistant *Vibrio* spp. infections. Given the species identification challenges in clinical settings, further work should also consider the use of genomic identification to replace current clinical methods or inform MLSA/MLST schemes.

Additionally, sample size was not adequate to individually evaluate each *bla*_CARB_ allele, and the AMR impacts of presumed conservative and non-conservative mutations may vary. Future studies could further investigate ß-lactamase secretion levels or CARB protein structure of isolates with various *bla*_CARB_ alleles as potential underlying causes of genotype–phenotype discordance.

### Conclusions

Advances in whole genome sequencing identification methods over the last decade, which utilize data from the entire genome, allowed for identification of *V. diabolicus* genomes that were previously misclassified and share many of the same AMR genes as known pathogenic species. Detection of previously misclassified *V. diabolicus* suggests that re-interpretation of earlier research may be warranted to better understand the implications for *Vibrio* epidemiology and AMR patterns in coastal systems. We characterized the genomic and phenotypic AMR patterns of a large sample set of *V. alginolyticus*, *V. cholerae*, *V. diabolicus*, and *V. parahaemolyticus* isolated from southern sea otters of coastal California as an understudied host. Through a novel genomic epidemiology approach, links between antimicrobial-resistant strains and isolation source were identified, as well as evidence that near-identical antimicrobial-resistant strains may persist over years in coastal marine systems. We have highlighted the discordance between phenotypic AMR and putative underlying genetic determinants in *Vibrio* species. Continued surveillance of phenotypes and genotypes is warranted to detect biologically relevant changes in AMR of *Vibrio* species over time. Through both phenotypic testing and alignment of *bla*_CARB_ genes in *V. parahaemolyticus*, we determined that specific allelic variants are more likely to result in ampicillin resistance, and that gene detection alone is not sufficient to predict AMR in *Vibrio* species.

## Data Availability

Metadata for the 489 *Vibrio* species genomes collected in coastal Alaska, California, and Washington from 2000 to 2019 have been made available with SRA accession numbers and BioSample numbers SAMN40178385–SAMN40178873 (Data Set S1) as part of the 100K Pathogen Genome Project.

## SUPPLEMENTAL MATERIAL

Data Set S1 aem.01808-24-s0001.xlsx MLSA/MLST results, genome quality metrics, and ampicillin susceptibility results for available genomes.

Supplemental figures aem.01808-24-s0002.docx Figures S1 and S2.

ASM does not own the copyrights to Supplemental Material that may be linked to, or accessed through, an article. The authors have granted ASM a non-exclusive, world-wide license to publish the Supplemental Material files. Please contact the corresponding author directly for reuse.
