# Supplementary material for: Antimicrobial resistance of Vibrio spp. from the coastal California system: discordance between genotypic and phenotypic patterns
Source: Appl Environ Microbiol. 2025 Feb 3;91(3):e01808-24. doi: 10.1128/aem.01808-24 (PMC11921324; doi:10.1128/aem.01808-24)
Supplement: Supplemental figures — Figures S1 and S2. [file aem.01808-24-s0002.docx]

# Supplementary Material


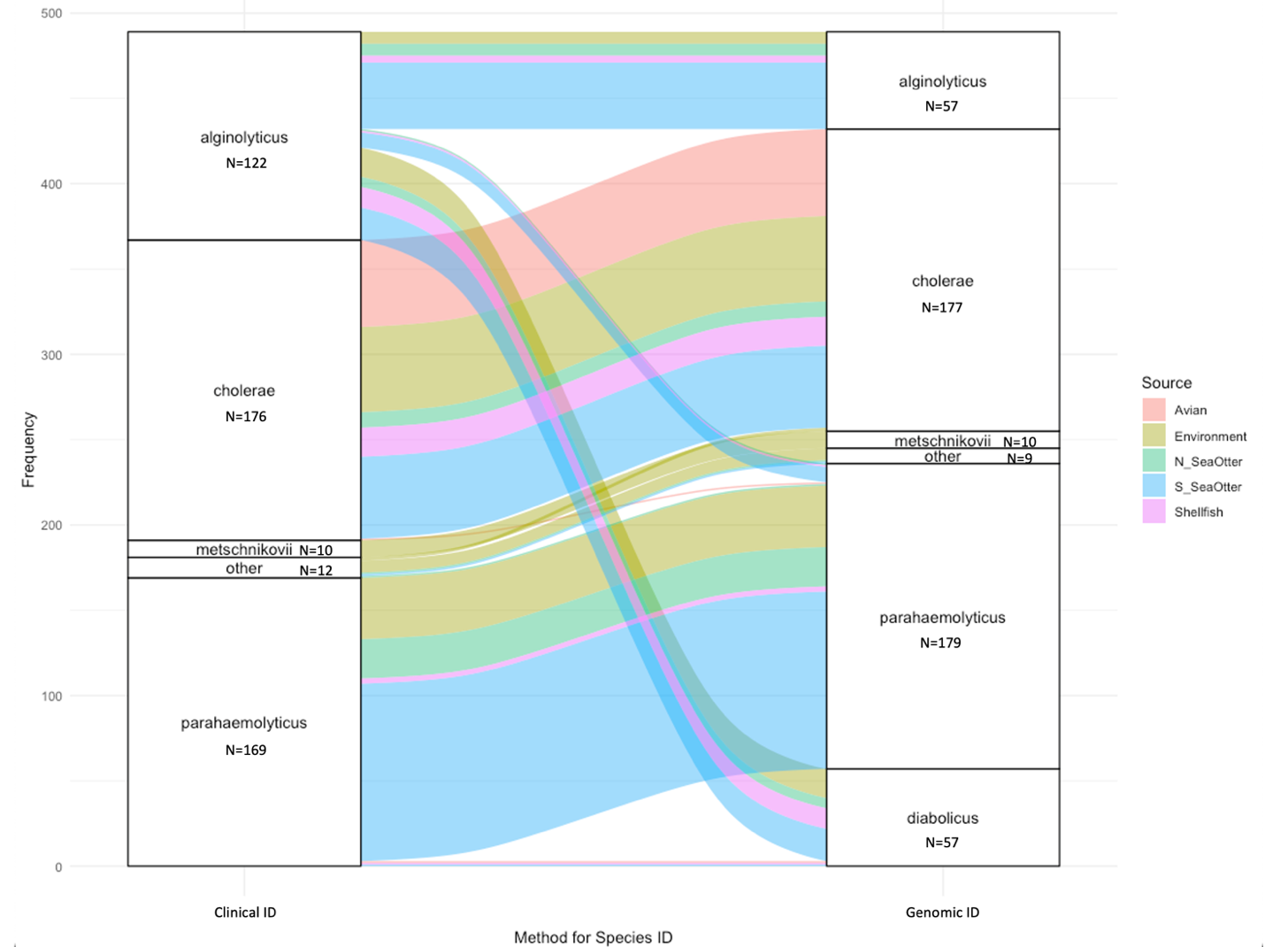


Supplemental Figure 1. Alluvial plot comparison of *Vibrio* species identification as labeled in the microbiology clinic versus using genomic methods for 489 isolates collected from various sources in coastal Alaska, California, and Washington between 2000-2019. While most isolates were corrected identified in the clinic (84%), the clinic methods were not able to identify *V. diabolicus* isolates.


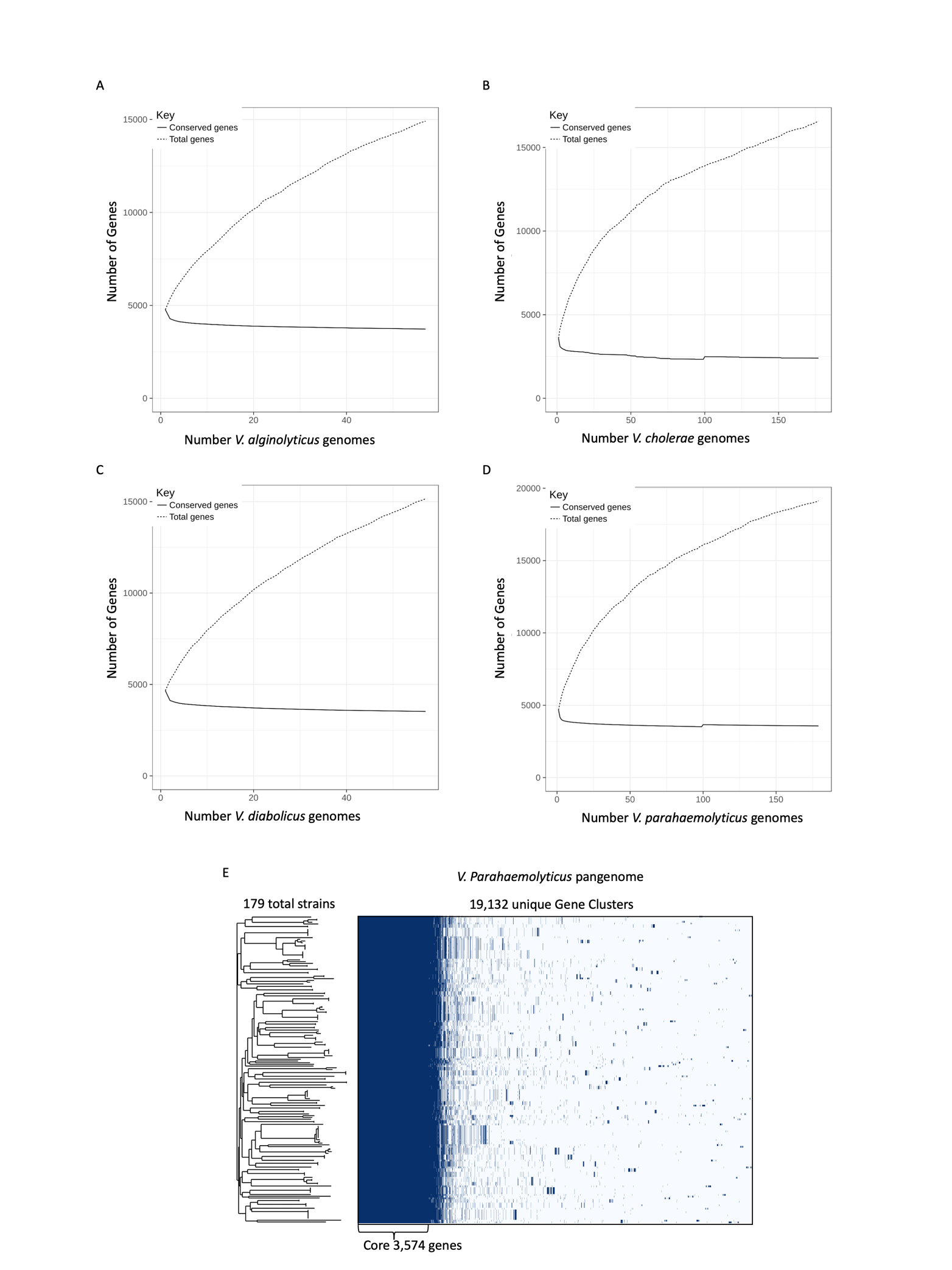


Supplemental Figure 2. Rarefaction curves for the pangenomes of A) *V. alginolyticus* (n=57), B) non-O1/O139 *V. cholerae* (n=177), C) *V. diabolicus* (n=57), and D) *V. parahaemolyticus* (n=179) collected from various sources in coastal Alaska, California, and Washington between 2000-2019. The total number of discovered genes in each pangenome continued to increase as additional genomes were added. E) Pangenome plot of *V. parahaemolyticus* (n=179) highlighting the small proportion of the total gene cluster pool (19,132) comprised of the core genome (3,574). Large accessory genomes were also observed for *V. alginolyticus*, *V. cholerae*, and *V. diabolicus* pangenomes. Blue blocks indicate the presence of a gene cluster (columns) in their respective genomes (rows).
